# A Deep Dive into the Botanical and Medicinal Heritage of *Taxus*

**DOI:** 10.3390/plants14101439

**Published:** 2025-05-11

**Authors:** Alex-Robert Jîjie, Dan Iliescu, Laura Sbârcea, Casiana Boru, Dalia Pătrașcu, Oana Andrada Iftode, Ionela-Daliana Minda, Ștefana Avram, Cristina-Maria Trandafirescu, Cristina Adriana Dehelean, Elena-Alina Moacă

**Affiliations:** 1 University Clinic of Toxicology, Drug Industry, Management and Legislation, Faculty of Pharmacy, “Victor Babes” University of Medicine and Pharmacy Timisoara, 2nd Eftimie Murgu Square, 300041 Timisoara, Romania; alex-robert.jijie@umft.ro (A.-R.J.); patrascu.dalia@umft.ro (D.P.); andradaiftode@umft.ro (O.A.I.); cadehelean@umft.ro (C.A.D.); alina.moaca@umft.ro (E.-A.M.); 2 Research Centre for Pharmaco-Toxicological Evaluation, Faculty of Pharmacy, “Victor Babes” University of Medicine and Pharmacy, 2nd Eftimie Murgu Square, 300041 Timisoara, Romania; 3 University Clinic of Surgical Semiology I and Thoracic Surgery, Faculty of Medicine, “Victor Babes” University of Timisoara, 2 Eftimie Murgu Square, 300041 Timisoara, Romania; dan.iliescu@umft.ro; 4 University Department of Drug Analysis, Environmental Chemistry, Hygiene, Nutrition, Faculty of Pharmacy, “Victor Babes” University of Medicine and Pharmacy, 2nd Eftimie Murgu Square, 300041 Timisoara, Romania; 5 Advanced Instrumental Screening Center, Faculty of Pharmacy, “Victor Babes” University of Medicine and Pharmacy Timisoara, 2nd Eftimie Murgu Square, 300041 Timisoara, Romania; 6 Faculty of Medicine, “Vasile Goldis” Western University of Arad, 86 Liviu Rebreanu Street, 310048 Arad, Romania; 7 University Department of Pharmacognosy, Faculty of Pharmacy, “Victor Babes” University of Medicine and Pharmacy Timisoara, 2nd Eftimie Murgu Square, 300041 Timisoara, Romania; daliana.minda@umft.ro (I.-D.M.); stefana.avram@umft.ro (Ș.A.); 8 Research and Processing Center for Medicinal and Aromatic Plants, Faculty of Pharmacy, “Victor Babes” University of Medicine and Pharmacy Timisoara, 2nd Eftimie Murgu Square, 300041 Timisoara, Romania; 9 Department of Pharmaceutical Chemistry, Faculty of Pharmacy, “Victor Babes” University of Medicine and Pharmacy Timisoara, 2nd Eftimie Murgu Square, 300041 Timisoara, Romania; trandafirescu.cristina@umft.ro

**Keywords:** *Taxus* spp., yew, *Taxus* aril, anticancer properties, therapeutic applications, phytocompounds, green medicine

## Abstract

The genus *Taxus* comprises a unique group of gymnosperms known for their botanical longevity, cultural significance, and exceptional pharmacological potential. This review explores the multifaceted profile of *Taxus* species, with a focus on their morphological traits, phytochemical composition, traditional uses, and therapeutic applications. Particular attention is given to taxanes, especially paclitaxel, which have revolutionized cancer treatment through microtubule-stabilizing mechanisms. In addition to well-established uses of the bark and leaves, the review synthesizes emerging research on the aril, a non-toxic and antioxidant-rich plant part, suggesting novel biomedical applications. By integrating ethnobotanical knowledge with contemporary pharmacological insights, this work underscores the enduring relevance of *Taxus* in traditional medicine while emphasizing its evolving role in modern drug discovery. The findings advocate for intensified interdisciplinary research and sustainable exploitation strategies to fully harness the genus’s therapeutic potential without compromising biodiversity.

## 1. Introduction

### 1.1. Historical and Cultural Significance of Taxus

The genus *Taxus*, commonly known as yew, includes a group of long-lived gymnosperms with a rich botanical and medicinal heritage. Recognized as one of Europe’s most ancient tree lineages, *Taxus* species are esteemed for their longevity and mythological significance in various cultural contexts, including Celtic, Greek, and Roman traditions [1,2,3]. Their durable wood and physiological resilience contributed not only to their practical applications in traditional societies but also to their spiritual symbolism of death, rebirth, and immortality. These attributes made yew trees prominent features of sacred spaces across Europe [4,5]. Ancient specimens of *Taxus* are often located in or near early Christian churchyards, prehistoric burial sites, and ritual landscapes. Notable examples include the Fortingall Yew in Scotland, estimated to be between 2000 and 5000 years old, and the Defynnog Yew in Wales, believed to be over 3000 years old, both found in churchyards likely built over pre-Christian sanctuaries. Other sacred sites with ancient yews include Kingley Vale in England, the churchyards of La Haye-de-Routot in France, and the Montejo de la Sierra reserve in Spain. The enduring association of yew trees with sacredness across pagan and Christian traditions demonstrates their role as living witnesses of spiritual continuity and transformation throughout European history [3,6,7,8,9,10].

### 1.2. Pharmacological Importance and Discovery of Taxanes

Botanically, *Taxus* species are of exceptional interest due to their phytochemical profiles, especially their production of taxanes, diterpenoid alkaloids that serve as precursors for anticancer drugs like paclitaxel (Taxol^®^), docetaxel (Taxotere^®^), and cabazitaxel (Jevtana^®^). The groundbreaking discovery of paclitaxel from *Taxus brevifolia* bark in 1965 by Wall and Wani marked a new era in cancer chemotherapy. Since its FDA approval in the early 1990s, paclitaxel has become an essential chemotherapeutic agent for treating ovarian, breast, and lung cancers [11,12]. It is currently listed on the World Health Organization’s List of Essential Medicines due to its clinical relevance and efficacy in cancer therapy [13,14]. Taxanes are regarded as potent antitumor agents, capable of inducing partial or complete remission in approximately 30–50% of cancer cases, depending on the stage at which therapy is initiated [15].

In addition to paclitaxel, other important compounds are docetaxel and cabazitaxel, synthesized semisynthetically from precursors like 10-deacetylbaccatin III. Docetaxel is extensively utilized in the treatment of various cancers, including breast and non-small cell lung cancers, and acts by promoting microtubule stabilization, similar to paclitaxel [16,17]. Cabazitaxel is primarily employed for treating hormone-refractory metastatic prostate cancer, showcasing effectiveness where other treatments have failed, thus diversifying therapeutic options [18,19]. Current research aims to refine taxane formulations by incorporating nanotechnology-based delivery systems and combinatorial therapies to counteract multidrug resistance and adverse reactions [20,21]. Such innovations have broadened the scope of taxane utility in clinical oncology, reflecting a paradigm shift in the application of phytochemicals in modern medicine.

### 1.3. Sustainability Concerns and Research Trends

Importantly, the sustainability of taxane sourcing has emerged as a critical issue. The overharvesting of *Taxus* bark and the species’ slow growth rate have led to legal protections and the exploration of alternative sources, including cell culture systems and synthetic biology approaches. In addition to extraction from natural sources, paclitaxel can also be obtained through semi-synthetic approaches, most notably by chemical conversion from 10-deacetylbaccatin III, a precursor compound abundant in *Taxus* needles, enabling more sustainable and scalable production strategies [22,23].

The global taxane market is projected to experience substantial growth, with detailed forecasts estimating continuous expansion from 2020 to 2029 across both generic and branded drug categories, various formulations (including nanoparticles and liposomes), and multiple cancer indications such as breast, ovarian, and prostate cancer. This trend is driven by rising global cancer incidence, innovation in formulation technologies, and broader accessibility across key healthcare systems in North America, Europe, and Asia-Pacific. These dynamics collectively underscore the strategic importance of taxanes in the global oncology pharmaceutical market [24]. This underscores the necessity of sustainable harvesting methods and innovative biosynthetic technologies.

The concentration of paclitaxel in the bark of *Taxus* species is typically around 0.007–0.01% [25]. Notably, the average paclitaxel content in the bark has been reported to be approximately 64% higher in male trees compared to female ones [26]. It is estimated that approximately one gram of paclitaxel can be extracted from three to four 60-year-old trees. From 1 kg of dried bark, roughly 50–150 milligrams of paclitaxel can be obtained, and an average tree yields around 10 kg of bark [25]. Global demand for paclitaxel continues to rise, with the estimated annual requirement for purified substance reaching approximately 250 kg. To treat 500 cancer patients, approximately 1 kg of purified paclitaxel is needed, requiring nearly 10 metric tons of bark and the harvesting of approximately 700 mature trees. On average, the production of 1 kg of purified Taxol demands between 7000 and 10,000 kg of bark, translating to the destruction of up to 750,000 trees annually to meet current therapeutic needs [27,28]. According to the National Cancer Institute (NCI), the extremely low natural content of paclitaxel in *Taxus* bark is insufficient to meet both market and research demands. The slow growth rate of *Taxus* trees, coupled with the high cost of extraction and the ecological implications of large-scale harvesting, renders this production method both economically burdensome and environmentally unsustainable [29].

Beyond the well-documented anticancer compounds from the bark and leaves, recent studies have highlighted the pharmacological potential of the *Taxus* aril, the fleshy, red, and non-toxic seed appendage [30]. The aril is rich in antioxidants, flavonoids, carotenoids, and mucilages. These bioactives are linked to a wide range of health benefits, including anticancer, hypoglycemic, hepatoprotective, and antimicrobial effects [25,30,31,32,33]. Early-stage investigations indicate that aril-derived compounds like rhodoxanthin possess anti-melanoma properties and may also serve as antioxidants and natural preservatives in food and cosmetic products [31,34]. Research on the phytochemistry and complex bioactivity of the *Taxus* aril is still in its early stages, with only a limited number of studies available in the scientific literature. Investigations of this plant part have only recently gained attention, emerging primarily in the last few years.

The dualistic nature of *Taxus*, as both a toxic and a therapeutic agent, highlights its complexity and relevance to both traditional and modern medicinal systems. While traditional applications employed extracts from various anatomical parts to treat fevers, respiratory ailments, and epilepsy, scientific inquiry has now validated many of these ethnobotanical uses through mechanistic pharmacological studies [35,36,37].

### 1.4. Scope of This Review

The scope of this review is to provide a comprehensive overview of the genus *Taxus*, by exploring its botanical characteristics, historical and ethnomedicinal significance, traditional therapeutic uses, phytochemical diversity, and the evolving pharmacological applications of its compounds. In addition, the review highlights emerging data on the nutritional and medicinal potential of the aril, the only non-toxic part of the plant, with the aim of expanding the future scientific research and understanding of this genus beyond its established anticancer applications.

## 2. Botanical Overview of *Taxus* Species

Species of the genus *Taxus* are exceptionally long-lived gymnosperms, with some individuals reaching up to 5000 years [1]. They are typically small to medium-sized trees, growing 10–30 m tall depending on species and environment [13]. Growth is slow, about 30 cm annually in young trees, and vertical growth generally halts after 100 years [38,39].

Branches are slender and flexible, remaining green for two years before turning reddish-brown [4]. Trunk diameters usually range from 2 to 4 m, occasionally exceeding 5 m in ancient specimens [33,40]. The bark is thin, reddish-brown, smooth, and exfoliates in scaly plates [33].

Leaves are evergreen, leathery, dark green above and lighter beneath, arranged pectinately or spirally. They are linear-lanceolate (1–4 cm long, 2–3 mm wide), with acuminate, cuspidate, or mucronate apices, slightly revolute margins, and a single midvein. They may be sessile or short-petiolate [13,33,40].

*Taxus* species reproduce sexually via seeds [1], which mature from October to November [26], though germination may take over 18 months [38]. The ovoid seeds (5–8 mm) are encased in a woody episperm and partially or fully surrounded by a red, fleshy aril, the plant’s only non-toxic part, consumed by wildlife for seed dispersal [26,33].

Yews are dioecious, with male and female cones on separate trees [33]. Flowering occurs from March to May, and wind pollination spans from September to April [26]. Male cones are pale yellow, ovoid, and located along branches, each with 8–14 microsporophylls bearing 4–8 pollen sacs. Female cones form at branchlet tips, accompanied by sterile scale-like leaves [40].

*Taxus* species generally prefer temperate forest habitats characterized by cool, moist, and shaded environments. They are typically found under the canopy of mixed or coniferous forests, often thriving on well-drained soils with moderate to high humidity. *Taxus baccata*, for instance, is commonly associated with limestone soils and calcareous substrates across Europe, while *T. wallichiana* occupies montane and subalpine zones (2000–3300 m) in the Himalayas, particularly on north-facing slopes with deep, moist soil profiles. *T. cuspidata* and *T. canadensis* show a marked preference for acidic, humus-rich forest floors in northeastern Asia and North America, respectively. Although adaptable, these species are sensitive to habitat disturbance and canopy removal, which have contributed to their population fragmentation and decline in several regions [26,39,41,42].

Due to their extreme longevity and shrinking natural distribution, *Taxus* species are legally protected in many regions. In China, they receive first-class protection; in India, their export is prohibited [43,44].

They are threatened by slow growth, low reproductive output, fragmented populations, climate change, illegal trade, and overharvesting for taxane production [2,13,45]. The genus is in decline and restricted mostly to isolated populations [4], prompting its inclusion on the IUCN Red List (Table 1) [38,46].

Conservation should integrate genetic and ecological studies to inform species management. Some populations exhibit unique genetic traits crucial for resilience and survival [42,48,49]. Biotechnological approaches can aid in sustainable harvesting and reduce pressure on wild populations [50].

## 3. Traditional Uses in Folk Medicine

*Taxus* species, particularly *Taxus wallichiana* and *Taxus baccata*, have a significant place in traditional medicinal systems and folk medicine due to their diverse therapeutic properties. These trees were traditionally valued for their bark, leaves, and arils, which were used in various medicinal preparations across different cultures, including those of Himalayan communities and Indigenous populations in Europe [35].

One of the primary traditional uses of *Taxus* is its application in treating respiratory ailments. For instance, the leaves and bark of *Taxus* are historically employed to relieve coughs, colds, and fever. In Ayurveda and Unani medicine, practitioners utilize it for its efficacy in addressing common cold symptoms and inflammation reduction, highlighting its role as an anti-inflammatory agent [51]. Additionally, the arils of *Taxus*, particularly in the Mediterranean region, have been used to produce medicinal wine, which is believed to possess restorative properties [30].

Moreover, studies show that compounds derived from *Taxus* exhibit potent antitumor activities, largely due to their capacity to inhibit cell division through interactions with cellular pathways. This is particularly true for paclitaxel, a compound derived from the bark of the Pacific yew (*Taxus brevifolia*), which has gained prominence in modern oncology as a chemotherapeutic agent. In traditional settings, extracts from the leaves and stems have been administered for cancer treatment, showcasing a long-held belief in their curative properties [37,52,53].

Beyond cancer and respiratory disorders, *Taxus* species are also recognized for their potential in managing diabetes and metabolic issues. There has been anecdotal evidence supporting the use of *Taxus* for controlling blood sugar levels, owing to various phytochemicals present within the plant [30,54,55,56,57].

The cultural significance of *Taxus* also extends to its use in rituals and as a protective talisman. In some Indigenous cultures, components of the tree are revered and used in traditional ceremonies aimed at promoting health, protection, and harmony within the community [3,35]. This ethnobotanical perspective underscores the integral role of *Taxus* not simply as a medicinal resource but also as a cultural symbol.

Although *Taxus* species were traditionally used in folk medicine, it is important to note that they were processed using various methods aimed at reducing their content of toxic compounds (taxines). However, due to the plant’s high inherent toxicity, the direct consumption of unprocessed *Taxus* material is unsafe and strongly discouraged nowadays. In traditional medicine, particularly in the Himalayan and South Asian systems (e.g., Ayurveda and Unani), practitioners developed empirical detoxification methods to reduce the toxic effects of *Taxus* species. A common method involved boiling the bark or leaves for extended periods (sometimes over 2–3 h), a process thought to degrade thermolabile toxic compounds such as taxines A and B, which are sensitive to heat and oxidation. In many traditional Himalayan preparations, *Taxus* bark powder was boiled with milk, honey, ghee (clarified butter), or jaggery (unrefined sugar), substances believed to bind or neutralize toxins while enhancing the bioavailability of beneficial compounds. These lipid- or sugar-rich vehicles were thought to either bind lipid-soluble toxins (like ghee) or buffer harsh effects and improve tolerability (as with honey and milk), while also contributing to the taste and stability of the preparation, reducing the free concentration of toxic compounds in the decoction. In some formulations, the plant material was fermented or sun-dried for several days, which may have promoted enzymatic or microbial degradation of unstable constituents [26,35,37,51,58,59,60,61].

Table 2 summarizes the traditional medicinal uses of various *Taxus* species across different regions of the world, reflecting their ethnobotanical significance and diverse applications in folk medicine. A more detailed version of Table 2 is available in the Supplementary Materials (Table S1).

## 4. Phytochemistry of *Taxus* Species

Research indicates significant interspecies variability in the percentage content of bioactive compounds, with certain species exhibiting markedly higher concentrations of specific bioactive compounds. This variability is associated with genetic, epigenetic, and environmental factors [62]. Over the years, various analytical methodologies have identified approximately 3000 diterpene alkaloids in the leaves and bark of *Taxus* species [25,63]. Additionally, around 500 flavonoids have been characterized, encompassing several classes, including flavones, biflavones, flavonols, flavonol-glycosides, dihydro-flavones, dihydro-flavonols, dihydro-flavonol-glycosides, flavanols, biflavanols, and chalcones [64]. Furthermore, other secondary metabolites have been documented, such as lignans [26], volatile compounds (including alcohols, alkanes, alkenes, organic acids, and terpenes) [37], phytosterols, phytoecdysteroids [65], and coumarins [66].

The bark of *Taxus* species serves as a significant reservoir of diverse phytocompounds, with taxanes emerging as the most notable group of diterpene alkaloids, recognized for their critical role in oncological therapies. Research has elucidated the presence of several valuable taxanes within the bark, including paclitaxel, cephalomannin, 10-deacetylpaclitaxel, 10-deacetylbaccatin III, baccatin III, and 7-xylosyltaxanes [67,68]. Notably, paclitaxel is esteemed for its bioactive properties and its essential role in both traditional medicine and the formulation of contemporary anticancer pharmaceuticals. Furthermore, investigations have led to the identification of novel compounds, such as taxusumatrin, a new diterpene alkaloid isolated from the bark of *Taxus sumatrana* [69]. In addition to taxanes, the bark of *Taxus* species is also characterized by the presence of phenolic compounds, which exhibit antioxidant and anticarcinogenic properties, exemplified by α-conidendrin, isolated from the bark of *Taxus yunnanensis* [70].

The chemical composition of *Taxus* leaves exhibits variability not only among different species but also within the same species, influenced by factors such as geographical location and cultivation methods. These variations in chemical composition are critical, as they significantly affect the bioactivity and medicinal properties of *Taxus* leaves [71,72]. Diterpene alkaloids, including paclitaxel, cephalomannin, and 10-deacetylbaccatin III, have been identified in the leaves [71]. Additionally, flavonoids represent another class of compounds that are abundantly present in *Taxus* leaves; their distribution, biological activities, and structural characteristics are of particular interest in phytochemical research. The complexity of flavonoid structures and their distribution across *Taxus* species have been the focus of investigation to enhance understanding of their pharmacological activities and to explore their potential applications in future research and development [64,66].

Metabolic variations of flavonoids in *Taxus* leaves have been investigated utilizing advanced analytical methodologies, specifically ultra-performance liquid chromatography coupled with electrospray ionization tandem mass spectrometry (UPLC-ESI-MS/MS). This analysis has elucidated significant differences in flavonoid content across various *Taxus* species [66]. Additionally, other bioactive compounds present in the leaves include volatile oils, which encompass constituents such as benzene propanenitrile, 1,4-dioxan-2,3-diol, and 3-bromo-3-methyl butyric acid. The identification of these specific volatile compounds, along with their demonstrated antimicrobial activities, offers critical insights into the potential applications of these plant extracts in diverse dermatological contexts [73,74]. Furthermore, the leaves are also comprised of steroids, lignins, and polysaccharides [57,75].

Research on male cones has identified the presence of various biochemical constituents, including polysaccharides, fatty acids, phenolic compounds, steroid derivatives, coumarins, and volatile compounds [74].

In the context of *Taxus* species seeds, a diverse array of phytochemicals has been documented, encompassing alkaloids, flavonoids, lignans, polysaccharides, and steroid derivatives, thereby underscoring the extensive chemical diversity inherent in these seeds. Notably, paclitaxel emerges as a pivotal bioactive compound, recognized for its significant pharmacological properties. Additionally, several alkaloids have been characterized within the seeds, including taxinin A, baccatin III, 9-deacetyltaxinin, 2-deacetyltaxinin, taxezopidin G, 2-deacetoxitaxynin J, and 2-deacetoxitaxuspin C. Furthermore, among the flavonoids detected in the seeds are naringenin, aromadendrin, galanin, epigallocatechin, and gallocatechin [71]. The seeds of *Taxus* demonstrate a distinctive fatty acid composition, characterized by the presence of taxoleic acid, a unique ∆5-olefinic acid. This specific composition serves to differentiate *Taxus* from other gymnosperms [76].

Historically, the arils of *Taxus* species have remained largely unexplored; however, recent phytochemical investigations have commenced, revealing their beneficial properties. The arils have been found to contain a diverse array of primary and secondary metabolites, including carotenoids, flavonoids, phenolic acids, terpenoids, vitamins, carbohydrates, micronutrients, macronutrients, lipids, and amino acids. This variety of phytocompounds contributes to the yet-to-be-fully elucidated medicinal, nutraceutical, and dermatological properties of the arils, positioning them as a possible valuable natural resource within the medico-pharmaceutical domain [31,32,34,77,78,79].

**Figure 1:**
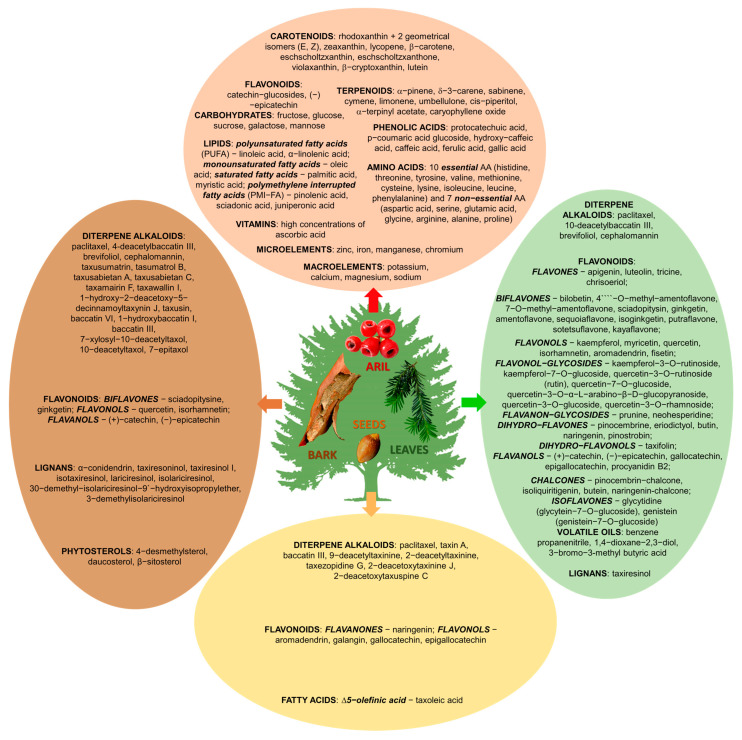
Schematic representation of the principal classes of primary and secondary metabolites found within the genus *Taxus*, alongside a delineation of the most notable compounds associated with each class. This illustration serves to elucidate the biochemical diversity inherent to *Taxus* species, highlighting the significance of these metabolites in ecological interactions and potential pharmacological applications. The primary metabolites, which include carbohydrates, proteins, and lipids, are essential for growth and development, while the secondary metabolites, such as alkaloids and flavonoids, play critical roles in defense mechanisms and have garnered interest for their therapeutic properties. The sources of the chemical composition data illustrated in this figure are detailed in the references below [25,26,29,31,32,34,37,51,57,64,65,66,67,68,71,77,78,79,80,81,82,83,84].

Figure 1 presents the considerable complexity and variability of the chemical composition across different plant parts of *Taxus* species, including the bark, leaves, seeds, and arils. This diverse chemical profile is fundamental to the various medicinal and toxicological attributes associated with *Taxus* species.

**Figure 2:**
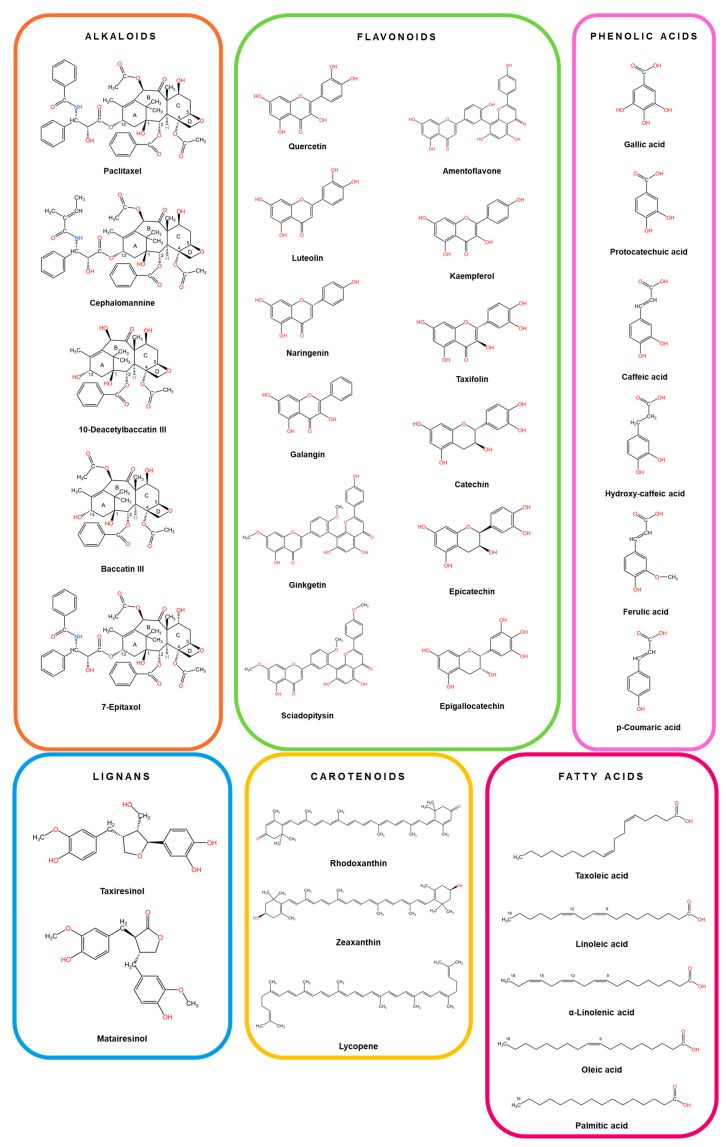
Chemical Structures of Key Compounds Found in *Taxus*. The chemical structures were created using KingDrawHD v1.4.5.-20230617 software.

Figure 2 presents the chemical structures of major bioactive compounds identified in different anatomical parts of *Taxus* species.

## 5. Pharmacological and Therapeutic Applications

### 5.1. Medicinal Benefits

The genus *Taxus* is well-documented for its medicinal properties, characterized by a range of bioactive compounds that exhibit diverse therapeutic effects. Numerous studies have elucidated the medicinal applications of *Taxus* species, particularly their potential for tumor suppression [55,68,85]. Additionally, these species demonstrate significant antibacterial activities [71,86], anti-inflammatory effects, and antioxidant properties [67], as well as potential applications in the management of diabetes [54,71,87], cardiovascular diseases [31], and neurological disorders [88,89].

**Figure 3:**
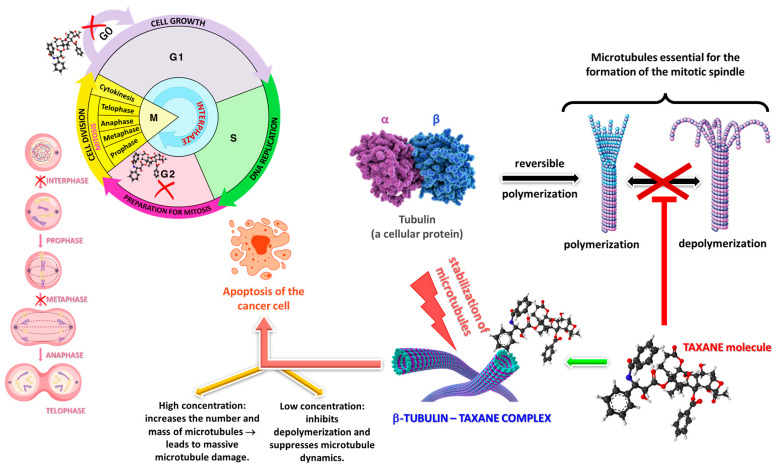
Mechanism of Action of Taxanes in Cancer Cells.

Paclitaxel, a key compound derived from *Taxus* species, is recognized as a potent anticancer agent utilized in the treatment of various malignancies, including sarcomas, melanomas, and carcinomas. Furthermore, it serves as an effective antineoplastic agent against breast, lung, and ovarian cancers [12]. Taxanes, particularly paclitaxel and docetaxel, exert their anticancer effects primarily by stabilizing microtubules, which disrupt normal mitotic spindle dynamics. This stabilization leads to cell cycle arrest in the M phase, ultimately triggering apoptosis in rapidly dividing cancer cells [53,90]. The mechanism of action involves binding to the β-subunit of tubulin, preventing its depolymerization, thereby inhibiting mitosis and other microtubule-dependent processes (Figure 3) [91]. Additionally, taxanes may impede the nuclear accumulation of the androgen receptor and other factors essential for tumor progression, particularly in prostate cancer [92,93]. While docetaxel demonstrates a higher affinity and antitumor activity compared to paclitaxel, both drugs play crucial roles in treating malignancies [94,95].

The leaves of *Taxus* species are particularly esteemed for their rich content of taxanes and flavonoids, which are integral to their medicinal efficacy. Empirical studies have substantiated the anticancer properties of aqueous extracts from the leaves of *Taxus* species, demonstrating their effectiveness in the treatment of pancreatic and lung cancers [96]. Additionally, these aqueous extracts have exhibited significant inhibitory effects on adenosine deaminase (ADA) activity in human cancerous gastric and colon tissues, potentially contributing to their anticancer mechanisms [97].

The cytotoxic activity of various components of *Taxus* species, including bark, leaves, and young branches, was systematically examined against a range of cancer cell lines, specifically HELA, T47D, MCF-7/HER2, LS174T, A549, MCF-7, and SMMC-7721. The findings indicate that *Taxus* species demonstrate substantial cytotoxic effects on cancer cells, thereby reinforcing their traditional application as medicinal plants possessing anticancer properties [86,98].

Flavonoids, including sciadopitysin, quercitrin, and ginkgetin, which are present in *Taxus* species, have been demonstrated to possess not only antitumor properties but also beneficial effects in the management of various conditions, such as osteoporosis, diabetic osteopathy, and Alzheimer’s disease [48,55,64,99].

The foliage of *Taxus* species is characterized by a high concentration of bioactive compounds, notably flavonoids, which are instrumental in imparting antioxidant properties [71,99]. These bioactive constituents have also been associated with analgesic and anti-inflammatory effects, substantiating their traditional medicinal applications. Moreover, empirical evidence indicates that *Taxus* species exhibit antipyretic and anticonvulsant properties [36,64].

The aril of *Taxus* species has been shown to exhibit antiproliferative and pro-apoptotic effects, primarily attributed to the carotenoid rhodoxanthin [34]. Isolated from the aril of *Taxus baccata*, rhodoxanthin has been associated with tumor growth inhibition and the modulation of antioxidant activity [77]. Furthermore, various tissues from *Taxus media*, including the aril, have been analyzed using metabolomics and antioxidant activity assessments, highlighting its potential for lowering blood glucose levels and treating kidney diseases, among other disorders [79]. Additionally, polymethylated fatty acids (PMI-FAs) in the aril confer immunomodulatory, antihypertensive, and hypolipidemic properties, and may also enhance memory performance [31,32,34,77,78].

Silver nanoparticles synthesized from the leaf extract of *Taxus wallichiana*, along with methanolic, chloroformic, ethyl acetate, and petroleum ether extracts, exhibit significant antibacterial activity against both Gram-positive bacteria (including *Staphylococcus aureus*, *Bacillus subtilis*, *Bacillus cereus*, and *Corynebacterium xerosis*) and Gram-negative bacteria (such as *Escherichia coli*, *Salmonella paratyphi* B, *Salmonella typhimurium*, *Pseudomonas aeruginosa*, *Pantoea agglomerans*, and *Yersinia pestis*) [75,86,100,101]. This antibacterial efficacy is primarily attributed to the presence of volatile compounds, including cis-3-hexen-1-ol, pentenyl-ethyl alcohol, and benzaldehyde, which are predominantly found in the leaves of *Taxus* species [71].

In addition to their antibacterial properties, *Taxus* species have also exhibited antifungal effects against various *Candida* species, including *Candida albicans*, *Candida tropicalis*, *Candida parapsilosis*, *Candida krusei*, and *Candida glabrata*, as well as against *Aspergillus brasiliensis* [80,102].

The antidiabetic properties of *Taxus* species have garnered considerable scholarly interest, with several species, including *Taxus chinensis* var. *mairei*, *Taxus cuspidata*, and *Taxus wallichiana*, exhibiting notable efficacy in traditional medicinal practices for diabetes management. The hypoglycemic effects of these *Taxus* species have been substantiated through both in vivo and in vitro investigations. These effects are characterized by mechanisms such as the impairment of insulin secretion, enhancement of glucose uptake in peripheral tissues, inhibition of carbohydrate-digesting enzymes, and the mimicking of insulin action [30,54,71,87].

The anti-asthmatic effect, a benefit derived from traditional medicine and corroborated by empirical studies, is noteworthy. Research indicates that species within the genus *Taxus* exhibit significant anti-asthmatic properties, which may confer therapeutic advantages in the management of asthma. This is achieved through the relaxation of bronchial smooth muscle and a reduction in bronchial hyperreactivity [68,103].

**Figure 4:**
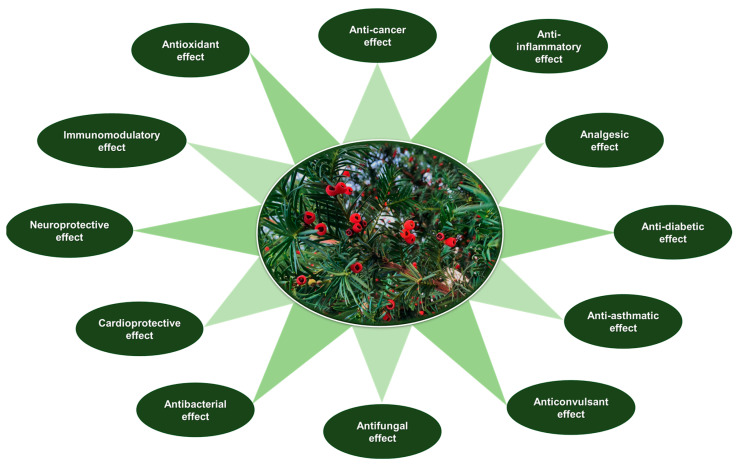
Schematic representation of the main pharmacological properties associated with *Taxus* species based on available in vitro and in vivo studies.

Figure 4 presents a comprehensive overview of the therapeutic effects attributed to *Taxus* species, particularly emphasizing their pharmacological significance.

*Taxus* species constitute a significant reservoir of bioactive compounds exhibiting a wide array of medicinal properties, including anticancer, anti-inflammatory, antioxidant, antibacterial, and antidiabetic activities. The complex chemical composition inherent to the *Taxus* genus underscores its relevance in both traditional and contemporary medicinal practices. Through a systematic combination of in vivo and in vitro investigations, researchers are progressively elucidating the therapeutic potential of *Taxus* species, thereby facilitating the advancement of novel pharmacological agents and therapeutic strategies (Table 3 and Table 4). The thorough examination of the medicinal attributes associated with *Taxus* species accentuates their critical role in modern medicine and underscores the imperative for further research aimed at fully harnessing their therapeutic capabilities.

**Figure 5:**
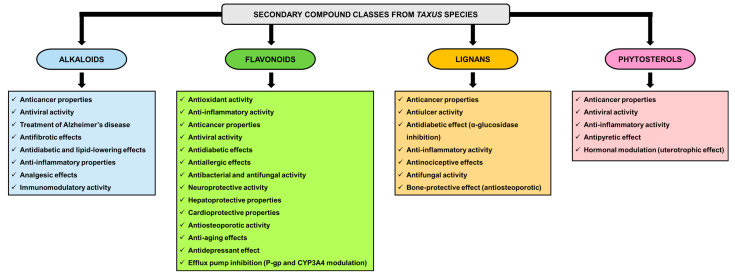
Schematic representation of the main pharmacological effects associated with the major classes of secondary metabolites identified in *Taxus* species.

Figure 5 summarizes the main bioactive classes identified in *Taxus* species and their associated therapeutic effects. The figure was developed based on the pharmacological activities attributed to each compound class, as detailed in Table 3.

### 5.2. Nutritional Value and Food Potential of Taxus Arils

The aril of *Taxus* species has garnered interest among researchers due to its nutraceutical potential, attributed to its rich chemical composition encompassing both primary and secondary metabolites. Recent studies have elucidated the phytochemical constituents of the aril, which include elevated concentrations of ascorbic acid, carotenoids, polyphenols, and volatile compounds. These components contribute to the aril’s antioxidant properties, thereby playing a role in mitigating oxidative stress. Furthermore, studies have demonstrated that arils are a valuable source of bioactive compounds and essential nutrients, characterized by their essential micro- and macroelements, amino acids, high-quality protein, and low levels of simple carbohydrates. This underscores the future potential usage of arils as a dietary component, particularly as a low-calorie snack, with 100 g of arils providing approximately 106 kilocalories according to researchers. Additionally, arils serve as a commendable source of zinc, potassium, chromium, and iron, with the consumption of 100 g fulfilling the recommended daily intake [31,32,34,77,78]. Although many other plant resources are known to be rich in antioxidant phytochemicals and essential nutrients, recent findings concerning the aril of *Taxus* species offer a particularly intriguing perspective. These discoveries significantly enhance our understanding of the aril’s therapeutic potential and provide scientific validation for its traditional applications in folk medicine. Furthermore, the identification of specific bioactive compounds supports its exploration as a complementary source of natural agents.

Tabaszewska et al. (2021) [31,32] were the first to comprehensively characterize the red arils (RAs) of *Taxus baccata*, suggesting that this underexplored plant part may represent a valuable source of nutraceutical compounds. The arils were found to be particularly rich in several key nutrients and bioactive molecules, which can be meaningfully contextualized by comparison with standard dietary sources. The most important ones include [32] the following:

- α-Linolenic acid (ALA), a key omega-3 fatty acid with cardioprotective and anti-inflammatory effects, was identified at ~1800 mg/100 g in *Taxus* arils. This level, while lower than in chia (17,500 mg/100 g) or flaxseed (22,800 mg/100 g) [150,151], greatly exceeds ALA levels found in conventional nuts such as walnuts (900 mg/100 g) or almonds (~1.5 mg/100 g) [152], and is almost absent in most fruits and vegetables.
- Sciadonic acid, a rare polymethylene-interrupted fatty acid (PMI-FA) with emerging anti-inflammatory potential, is present at ~276 mg/100 g in the aril, a feature not shared by typical food plants [32].
- Total protein content in the aril (~9.5%) surpasses that of most fruits (e.g., avocado: 2%, banana: 1.1%) and aligns with levels seen in legumes like cooked quinoa (~8%) or green peas (5.4%) [153,154,155,156]. More importantly, the essential amino acid (EAA) to total amino acid ratio of 40.5% is on par with high-quality proteins such as egg (43%) or soy (36–38%) [32,157].
- Mineral content is also notable. The potassium concentration (~500 mg/100 g) exceeds that of banana (358 mg/100 g) [154] and compares favorably with avocado (485 mg/100 g) [153] and sweet potato (337 mg/100 g) [158]. The iron content (3.0 mg/100 g) surpasses that of spinach (1.05 mg/100 g) [159], but is lower than that in lentils (7.1 mg/100 g) (https://fdc.nal.usda.gov/food-details/2644283/nutrients (accessed on 4 May 2025)).
- Rhodoxanthin, a rare red carotenoid scarcely found in conventional crops, is present in *Taxus* arils at ~760 µg/100 g, offering antioxidant potential distinct from classical carotenoids like β-carotene (carrot: 8500 µg/100 g), lycopene (tomato: 3100 µg/100 g), or capsanthin (red pepper) [32,160].

When considered collectively, these traits suggest that *Taxus* arils combine features of fruits with nutrient densities and functional compounds typically associated with seeds and medicinal plants. While the current evidence is primarily compositional, the presence of rare lipid mediators, essential nutrients, and bioactive pigments provides a strong rationale for continued investigation of the aril’s nutraceutical potential.

Research indicates that aril juice may play a preventive and ameliorative role in Alzheimer’s disease by influencing various critical biological processes associated with neuronal degeneration. These processes include neuronal apoptosis, amyloid fiber formation, oxidative stress, T-cell co-stimulation, inflammatory response, and insulin secretion [82,88,149].

Further scientific evidence supporting the nutraceutical potential of *Taxus* species is provided by a patent that describes the utilization of cultured cambial cells and *Taxus* stem procambium to formulate a functional beverage. In this process, 200 milligrams (mg) of cultured cells were dissolved in 96 milliliters of water. Subsequently, 500 mg of vitamin C was incorporated as an additive, along with 1 g of citric acid and 1 g of oligosaccharides to enhance flavor. Additionally, 0.05 g of sodium benzoate was included as a preservative. Purified water was then added in sufficient quantity to yield a total volume of 100 mL of the functional beverage [161].

**Figure 6:**
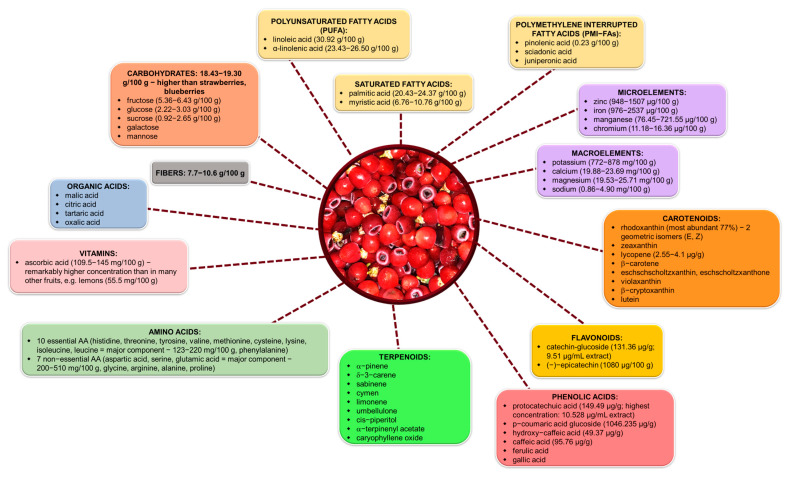
The biodiversity of primary and secondary metabolites in *Taxus* aril with potential nutraceutical applications. The sources of the chemical composition data illustrated in this figure are detailed in the references below [31,32,34,77,78,79,84].

Figure 6 provides a detailed overview of the nutritional and bioactive components found in *Taxus* arils, emphasizing their health-promoting properties. It categorizes arils’ composition into carbohydrates, fibers, organic acids, vitamins, fatty acids, amino acids, terpenoids, carotenoids, flavonoids, phenolic acids, macroelements, and microelements. These compounds collectively contribute to arils’ antioxidant, anti-inflammatory, and nutritional benefits.

The arils of *Taxus* species exhibit a complex chemical composition characterized by the presence of bioactive compounds, essential nutrients, and antioxidants, thereby positioning them as promising candidates for nutraceutical applications. The reviewed studies offer significant insights into the diverse array of compounds found within the arils and their associated potential health benefits. This underscores the necessity for further research aimed at fully harnessing the nutraceutical potential of these compounds.

The prospective use of *Taxus* arils as a dietary source of antioxidants remains a subject of ongoing scientific scrutiny and debate. Although preliminary analyses have revealed the presence of flavonoids, carotenoids, and other phenolic constituents in the arils, their nutritional and functional profiles are not yet as thoroughly characterized as those of commonly accepted antioxidant sources such as turmeric (*Curcuma longa* L.), green tea (*Camellia sinensis* Knutze), or rosemary (*Rosmarinus officinalis* L.) [162,163]. Furthermore, the relatively limited availability of arils and the narrow geographical distribution of *Taxus* species further restrict their viability as widespread food additives or nutraceuticals. As such, while the antioxidant potential of arils is scientifically intriguing, their incorporation into the food industry must be approached with caution, requiring deeper toxicological assessments, standardization of bioactive content, and comparative efficacy studies against established botanical antioxidants.

### 5.3. Dermatological Applications

While the therapeutic efficacy and pharmacological mechanisms of taxanes, particularly paclitaxel and its analogues, are extensively documented and well established in oncological applications, it must be emphasized that many of the other bioactive constituents derived from *Taxus* species remain in the experimental phase. Compounds such as flavonoids, lignans, and phenolic acids, though exhibiting promising in vitro antioxidant and anti-inflammatory properties, have not yet undergone the rigorous clinical validation necessary to support formal therapeutic or dermatological claims. As such, any reference to these compounds in the context of skin health or topical application should be interpreted as preliminary and exploratory, warranting further pharmacological and toxicological investigation.

It is crucial to underscore that, aside from the arils, which are the only non-toxic part of the plant, the remaining organs of *Taxus* species (e.g., leaves, bark, and seeds) contain potent toxic alkaloids, the difference between the therapeutic and toxic dose being very small. This inherent toxicity raises concerns regarding their direct use in formulations. Consequently, any potential dermatological applications involving extracts from toxic parts of the plant must be approached with strict caution. Such uses would necessitate comprehensive pharmaceutical documentation, toxicological profiling, and regulatory approval to ensure safety and compliance with legal standards.

The partial elucidation of the biodiversity of valuable phytocompounds identified in the arils of *Taxus* species [31,32,34,78] has prompted researchers to explore the potential applications of these arils in oncologic dermatology. A study utilizing the murine malignant melanoma model B16F10 demonstrated the properties of the retro-structured carotenoid, rhodoxanthin, isolated from the arils of *Taxus baccata*. This compound exhibited significant inhibitory effects on tumor growth and modulated antioxidant activity against murine malignant melanoma [77]. The antioxidant activity of rhodoxanthin is particularly pertinent to dermatology, as antioxidants are essential for protecting the skin from oxidative stress and mitigating premature aging [164]. Furthermore, the antitumor properties of this compound are of considerable interest in the context of treating malignant skin conditions, including skin cancer [165].

An in vivo study examining animal skin tissues elucidated the beneficial properties of *Taxus cuspidata* extract in the prevention and treatment of various dermatological conditions, including melanin deposition, oxidative stress, inflammation, and allergic reactions. Evidence indicates that the essential oil primarily exerts its antioxidant effects by enhancing the expression of key antioxidant enzymes, specifically superoxide dismutase (SOD) and glutathione peroxidase 4 (GPX4). Furthermore, the extract has exhibited antiallergic properties, as evidenced by a reduction in histamine release and modulation of pro-inflammatory cytokines, including tumor necrosis factor-alpha (TNF-α) and interleukin 1-beta (IL-1β), suggesting its potential efficacy against skin allergies. Additionally, the extract has been shown to diminish melanin deposits, thereby exerting a depigmenting effect through the inhibition of tyrosinase, a critical enzyme in melanin synthesis. These newly identified pharmacological effects of *Taxus* species, alongside their established antitumor properties, indicate a promising expansion of clinical applications within the dermatological domain [166].

Unexpectedly and somewhat controversially, the field of dermato-cosmetics has witnessed the emergence of two notable patents involving *Taxus* tissues:

1. A cosmetic composition incorporating cultured cells from the cambial or procambium regions of *Taxus* stems: This invention pertains to a formulation exhibiting antioxidant, anti-inflammatory, and anti-aging properties. It comprises one or more cell lines derived from the cambial or procambium tissues of *Taxus* stems, alongside their respective extracts, lysates, and culture media. The authors of this invention have endeavored to formulate compositions grounded in natural compounds that demonstrate significant antioxidant and anti-inflammatory efficacy. The inventors have established that cell lines derived from *Taxus* cambium and *Taxus* procambium, as well as their extracts, possess remarkable capabilities to mitigate inflammation and impede the aging process of the skin, which constitutes the core premise of this invention. The extract and cell line culture medium derived from the cell lines described in the present invention demonstrate a significant capacity to inhibit the synthesis of matrix metalloproteinase 1 (MMP-1), exhibiting effects comparable to those of retinoic acid, which is widely recognized for its potent anti-aging properties. This finding implies that the invention effectively mitigates collagen degradation, thereby contributing to the prevention of skin aging and the reduction of wrinkles, positioning it as a valuable candidate for anti-aging applications. Furthermore, the invention has been shown to inhibit melanogenesis in mouse melanoma cells, indicating its potential as a depigmenting agent [161]. The outcomes presented in this patent hold considerable significance, with the inventors having successfully developed functional formulations based on these findings.

Table 5 presents an overview of dermato-cosmetic formulations derived from patents that incorporate *Taxus* cambial and procambial cell extracts. The formulations include a body lotion and a cream, both of which utilize the beneficial properties of *Taxus* cambium and procambium. The body lotion formulation is designed to provide hydration and nourishment to the skin, leveraging the unique bioactive compounds found in *Taxus* extracts. Similarly, the cream formulation aims to enhance skin texture and promote overall skin health. The table systematically categorizes these formulations, highlighting their key ingredients and potential dermatological benefits, thereby illustrating the innovative use of *Taxus*-derived extracts in dermatology.

2. Goat’s milk soap incorporating *Taxus chinensis* extract: This invention pertains to a daily cleansing product, specifically a goat’s milk soap infused with *Taxus chinensis* extract. The formulation of the soap is as follows: 3–5 g of *Taxus chinensis* extract, 20–28 g of fresh goat’s milk, 0.8–1.2 g of virgin olive oil, 0.8–1.2 g of palm oil, 0.8–1.2 g of coconut oil, 0.8–1.2 g of mustard oil, and 45–50 g of soap base. The extract of *Taxus chinensis* is known to contain various skin-protective nutrients, while fresh goat’s milk is recognized for its skin-whitening properties. This soap formulation is designed to effectively cleanse the skin, promote whitening and moisturization, and retain moisture, thereby mitigating the risk of intracellular water loss [167].

**Figure 7:**
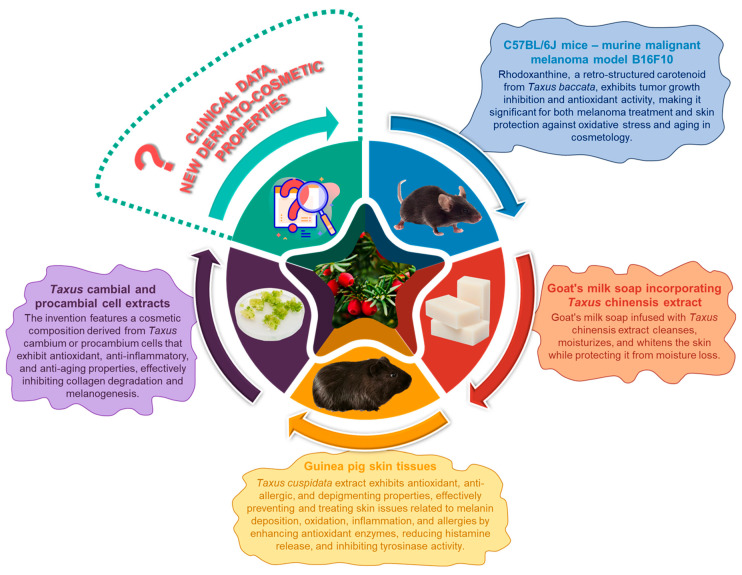
Overview of existing studies on the dermatological implications of *Taxus* species, highlighting the early-stage research and potential applications [77,161,166,167].

Figure 7 illustrates the limited but promising research on the role of *Taxus* species in dermatology. Current studies demonstrate the diverse applications of *Taxus* extracts in skin health and dermatologic formulations. The use of cambial and procambial cell extracts from *Taxus* showcases potent antioxidant, anti-inflammatory, and anti-aging properties, effectively preventing collagen degradation and reducing melanin synthesis. In murine models, rhodoxanthine, a carotenoid derived from *Taxus baccata*, shows tumor growth inhibition and antioxidant effects, suggesting applications in melanoma treatment and skin protection against oxidative stress. Another formulation includes goat’s milk soap infused with *Taxus chinensis* extract, providing cleansing, moisturizing, and whitening benefits while protecting the skin from moisture loss. Additionally, studies using guinea pig skin tissues reveal that *Taxus cuspidata* extracts offer antiallergic, depigmenting, and anti-inflammatory benefits, mitigating oxidative damage and regulating tyrosinase activity. Despite these findings, the field of dermatology utilizing *Taxus* species remains in its early stages, underscoring the necessity for additional clinical research and comprehensive exploration of the potential applications of *Taxus* extracts in dermato-cosmetic formulations.

Overall, the exploration of the implications of *Taxus* species in dermatology remains in its nascent stages, necessitating further research to elucidate the true potential of the genus *Taxus* for prospective applications in this domain.

## 6. Conclusions and Future Directions

The genus *Taxus*, with its intricate interplay of phytochemistry, ethnobotany, and pharmacology, remains a cornerstone in the realm of medicinal botany. Through this review, the multifaceted value of *Taxus* species has been presented,. The extensive repertoire of bioactive compounds, notably taxanes and flavonoids, validates the genus as a prolific source of therapeutic agents, particularly for cancer treatment, metabolic disorders, inflammatory diseases, and neurodegenerative conditions. Of equal significance is the recent emphasis on the aril of *Taxus*, a traditionally overlooked plant component that is now gaining attention for its antioxidant, antimicrobial, and anticancer properties. Moreover, despite the promising pharmacological potential of the *Taxus* aril, current scientific understanding remains limited due to the scarcity of studies and the preliminary nature of existing data. Future investigations are essential to validate these early findings and to elucidate the mechanisms of action, safety profiles, and clinical applicability of aril-derived compounds.

Despite the considerable strides in taxane isolation and semi-synthesis, the ecological burden of paclitaxel extraction underscores an urgent need for sustainable alternatives. Advances in synthetic biology, plant cell culture technologies, and endophytic fungi-mediated biosynthesis present promising avenues for scalable and eco-friendly production of taxanes. Furthermore, the ethnopharmacological validation of traditional uses opens new possibilities for developing plant-based therapeutics, particularly in the management of respiratory, hepatic, and metabolic ailments.

Future research should prioritize the following directions:

- Exploration of underutilized plant parts, such as arils, to uncover novel bioactives with pharmacological potential.
- Development of green synthesis methods for taxanes, including metabolic engineering in microbial systems and tissue culture optimization.
- Investigation into combinatorial therapies, leveraging taxane synergy with other plant-derived compounds or modern drug delivery platforms, especially nanocarriers.
- Clinical translation of lesser-known compounds, such as biflavonoids and polymethylated fatty acids, for use in oncology, neurology, and immunology.
- Integrative omics approaches to map species-specific metabolic pathways and understand interspecies variability in phytochemical content.

By elucidating the intricate interactions between the chemical constituents of *Taxus* species and their corresponding biological effects, future research may facilitate the development of innovative applications that harness both the traditional knowledge associated with these plants and the rigor of contemporary scientific validation. This dual approach not only honors the historical significance of *Taxus* in various therapeutic contexts but also ensures that its potential is substantiated through empirical evidence. Such investigations could lead to the identification of novel bioactive compounds and their mechanisms of action, thereby enhancing the utility of *Taxus* in modern medicine.

In conclusion, *Taxus* species represent not only a botanical legacy with deep cultural roots but also a bioscientific frontier with vast unexplored therapeutic horizons. Sustained interdisciplinary research is essential to unlock their full medicinal potential while ensuring conservation and ecological responsibility.

## Figures and Tables

**Table 1:** IUCN Red List Status and Threats for Selected *Taxus* Species Globally [47].

Species	IUCN Status	Geographic Distribution
*Taxus wallichiana*	Endangered	Himalayas: Afghanistan, India, Nepal, Bhutan, southern China
*Taxus chinensis*	Endangered	Southern China (Yunnan, Guizhou, Sichuan), northern Vietnam
*Taxus calcicola*	Vulnerable	China (Yunnan, Guizhou)
*Taxus baccata*	Least concern	Europe, North Africa, Western Asia
*Taxus brevifolia*	Near threatened	Pacific Northwest (USA: California to Alaska), British Columbia
*Taxus canadensis*	Least concern	Eastern Canada, Northeastern USA (Appalachians, Great Lakes region)
*Taxus mairei*	Vulnerable	Southern and eastern China, Vietnam, Taiwan
*Taxus contorta*	Endangered	Western Himalayas (Pakistan, India, Nepal)
*Taxus floridana*	Critically endangered	Northern Florida (Gadsden and Liberty Counties)
*Taxus globosa*	Endangered	Mexico and Central America (Sierra Madre Oriental)
*Taxus cuspidata*	Least concern	Northeast China, Korea, Japan, Russian Far East

**Table 2:** Traditional Uses of *Taxus* Species Around the World.

Species and Region:	Plant Part Used:	Administration:	Traditional Uses:	References:
*Taxus wallichiana* (India—Bhotiya tribe)	Bark	Tea	✓ to keep the body warm; ✓ treatment of hemorrhoids.	[26]
		Decoction	✓ treatment of muscle and joint pain, and rheumatism.	
		Decoction with jaggery	✓ treatment of hysteria.	
		Paste	✓ for the healing of fractured bones; ✓ treatment of headaches.	
	Young branches	Tincture	✓ prevention of headaches, dizziness, diarrhea, and weak pulse.	
		Decoction	✓ treatment of tuberculosis.	
	Leaves	Decoction/juice	✓ for liver disorders; ✓ treatment of asthma, cancer, and bronchitis.	
		Powder	✓ treatment of asthma, bronchitis, hiccups, epilepsy, diarrhea, and headaches.	
		Extract/juice	✓ for the treatment of cuts, wounds, and boils (external use); ✓ sedative; ✓ as an antidote against snake bites and scorpion stings.	
*Taxus wallichiana* (India)	Bark, seeds	Extract (oral)	✓ treatment of intestinal parasites.	[37]
	Bark	Tea	✓ treatment of high blood pressure, asthma, headaches, dizziness, and tumors.	
		Tea	✓ treatment of colds, asthma, arthritis, dizziness, and tumors.	
		Paste	✓ for healing fractured bones, treating headaches, and hemorrhoids.	
		Tea mixed with salt and ghee	✓ treatment of high blood pressure and cancer.	
		Paste mixed with egg yolk	✓ used as a plaster for healing fractured bones.	
		Decoction	✓ treatment of cancer.	
	Leaves	Juice from the leaves	✓ treatment of wounds, cuts, and boils.	
		Decoction	✓ treatment of asthma, bronchitis, and colds.	
		Decoction of leaves with honey	✓ treatment of fever, flatulence, epilepsy, and asthma.	
		Tea	✓ treatment of asthma and fever.	
	Bark, leaves	Tea	✓ treatment of cancer and tumors; ✓ treatment of swellings, congestion, cough, and asthma; ✓ used as a contraceptive measure.	
	Young branches	Tincture	✓ treatment of headaches, dizziness, weak pulse, diarrhea, and severe biliary disorders.	
	Stem	Decoction	✓ treatment of tuberculosis.	
	The entire plant	—	✓ treatment of cancer, jaundice, heart disorders, headaches, renal and digestive disorders; ✓ exhibits antispasmodic, laxative, and antirheumatic effects.	
*Taxus baccata* (India—Pauri district, Uttarakhand)	Bark, leaves	—	✓ treatment of headaches, bone fractures, cancer, asthma, bronchitis, epilepsy, arthritis, and snake bites.	[60]
*Taxus contorta*, *Taxus mairei*, *Taxus wallichiana* (Hindu Kush-Himalayan region)	Arils	Consumed as is	✓ used as a snack.	[35]
*Taxus contorta*, *Taxus mairei*, *Taxus wallichiana* (Hindu Kush-Himalayan region)	Leaves	Juice from leaves with honey	✓ cleanses the respiratory tract and helps treat cough and colds.	
*Taxus contorta*, *Taxus mairei*, *Taxus wallichiana* (Hindu Kush-Himalayan region)	Leaves	Juice or decoction (oral)	✓ treatment of diarrhea, indigestion, stomach pain, and liver disorders.	
	Aril	Consumed as is	✓ has carminative, expectorant, and stomachic properties.	
	Leaves, bark	Juice	✓ treatment of fever, low pulse, and cancer.	
	Bark	Tea	✓ used to enhance virility.	
*Taxus wallichiana* (Hindu Kush-Himalayan region)	Leaves, bark	Paste with honey	✓ treatment of bronchitis, asthma, and other respiratory problems.	
*Taxus contorta*, *Taxus wallichiana* (Hindu Kush-Himalayan region)	Leaves	Leaf juice (oral)	✓ treatment of headaches.	
	Bark	Paste (external use)	✓ applied on the forehead for headache relief.	
		Decoction	✓ treatment of muscle and joint pain, and rheumatism.	
*Taxus wallichiana*	—	Decoction, tea, juice	✓ treatment of colds, cough, respiratory infections, indigestion, and epilepsy.	[51,61]
	—	Poultice	✓ treatment of burns and infected wounds.	
	Bark	Paste	✓ treatment of bone fractures and headaches.	
	Bark, leaves	Steam baths	✓ treatment of rheumatism.	
	Stem	Decoction—in Pakistan	✓ treatment of tuberculosis.	
	Bark, leaves	Unani Medicine	✓ sedative, aphrodisiac; ✓ treatment for bronchitis, asthma, epilepsy, snake bites, and scorpion stings.	
	Young branches	Ayurvedic tincture	✓ used to treat severe biliary disorders, dizziness, weak pulse, cold extremities, headaches, and diarrhea.	
*Taxus baccata*	—	Asturias, León	✓ treatment of rheumatism, arthritis, liver disorders, and urinary tract disorders.	[5]
	Aril	Aril pulp—syrup (Northern Spain)	✓ treatment of pulmonary disorders.	
*Taxus wallichiana* (Indian Ayurvedic Pharmacopoeia)	Leaves	Powder	✓ with antirheumatic, anticatarrhal, and insecticidal action; ✓ wound healing; ✓ treatment of tumors, dermatoses, and helminthiasis.	[26]
*Taxus baccata* (Ukraine, Bukovina)	Bark	Bark decoction	✓ treatment against rabies.	[4]
*Taxus baccata* (Narew River, Northeast Poland)	Bark	Bark powder	✓ treatment against rabies.	
*Taxus baccata* (Central Balkan Peninsula)	Bark	Crushed bark	✓ treatment of rabies, epilepsy, and tuberculosis.	
*Taxus baccata* (Poland—Wólka Jagielczynska village, Częstochowa)	Bark	Infusion with milk or fumigation of the wood	✓ treatment of rabies.	

**Table 3:** Main Secondary Metabolites Isolated from *Taxus* Plant Parts and Their Pharmacological Properties.

Plant Part	Chemical Compound		Pharmacological Properties		References
**ALKALOIDS**					
Bark, leaves, roots	Paclitaxel (Taxol A) 0.007–0.01%		Anticancer effect: widely used to treat various types of cancer (lung, breast, blood, liver, brain, kidney, prostate, colon, cervical, gastric, pancreatic, Kaposi’s sarcoma). Antiviral effect (HIV, SARS-CoV-2). Low doses: treatment of liver, lung, and kidney fibrosis (0.3 mg/kg, 2×/week), coronary artery restenosis (1.3–10 µg/mm^2^), Alzheimer’s disease.		[25,27,28,29,104]
Leaves	10-Deacetylbaccatin III (1.76%)		Ethyl acetate extract: benefits against insulin resistance associated with inflammation and high-fat diet in C57BL/6 mice; significantly improves glucose uptake in skeletal muscle cells with inflammation-induced insulin resistance; dose-dependent reduction in lipid accumulation; decreased LDL, triglycerides, and total cholesterol, increased HDL; reduction of pro-inflammatory cytokines (TNF-α, IL-6, IL-1β); enhanced GLUT4 expression and distribution; no cytotoxicity at effective dose.		[56,81]
Leaves	Brevifoliol (7.59%)				[56]
Bark	4-Deacetylbaccatin III		Anti-inflammatory, analgesic effect (in vitro).		[51]
Bark	Tasumatrol B		Pronounced anti-inflammatory effect, remarkable analgesic effect.		[25,51,65]
Bark	Taxusabietane A		Significant anti-inflammatory effect (in vivo, 5–10 mg/kg), via 5-lipoxygenase inhibition at IC_50_ = 57 ± 0.31 μmol/L.		[25,51,65]
Bark	Taxusabietane C		Significant anti-inflammatory effect (IC50 = 69 ± 0.31 μmol/L).		[65]
Bark	Taxamairin F		Significant anti-inflammatory effect (IC50 = 73 ± 0.14 μmol/L).		[65]
Bark	Taxawallin I		Methanolic extract: strong cytotoxic activity in vitro against various cancer cell lines (A498, MDR 2780AD, NCI-H226, HepG2).		[25,65]
Bark	1-Hydroxy-2-deacetoxy-5-decinnamoyltaxinine J		Significant, dose-dependent anticancer effect against Colo 320DM, MCF-7, KB PA1, WRL-68 cell lines; immunomodulatory activity (1 µg/mL), enhances concanavalin A effects.		[25,65]
Bark	Taxusine		Ethanolic extract: anti-inflammatory, antinociceptive properties.		[68]
Bark	Baccatin VI				
Bark	1-Hydroxybaccatin I				
**FLAVONOIDS**					
Branches, leaves	Flavone	Apigenin	Antioxidant (0.5–32 µg/mL—strong inhibition of reactive oxygen species); anticancer (synergistic with paclitaxel in HeLa cervical carcinoma cells, reducing cell viability by 29% and increasing apoptosis by 24%); antidepressant (in mice at 50 mg/kg—increased immobility, swimming, and climbing time); anti-inflammatory (50–200 µmol/L—inhibits nitric oxide production and phagocytosis); potential protective effect in UV-induced cutaneous tumors.		[64,66,105,106,107,108]
Branches		Luteolin	Antioxidant (0.5–32 µg/mL); anticancer (synergistic effect with paclitaxel in human colorectal carcinoma cells); anti-inflammatory.		[64,105,106,108]
Leaves		Tricin	High potential as a functional agent in glycemic control; anti-inflammatory and anticancer effects.		[66,109]
Leaves		Chrysoeriol	Exhibits anti-inflammatory, anticancer, and anti-osteoporotic activity.		[66,108]
Leaves, branches	Biflavone	Bilobetin	Antibacterial and significant antifungal activity (against *Alternaria alternata*, *Cladosporium oxysporum*, *Fusarium culmorum*—median effective doses ED₅₀ = 14, 11, and 17 mmol/L, respectively; inhibited growth of *C. oxysporum* and *F. culmorum* at 100 mmol/L); acts as an efflux transporter inhibitor of P-glycoprotein (P-gp) and CYP3A4, enhancing the oral absorption of paclitaxel by limiting P-gp activity at a concentration of 50 mg/mL; concurrently, it reduced both the expression and activity of CYP3A4 at 100 µg/mL.		[64,110]
Leaves		4‴-O-methyl amentoflavone	Anticancer effect (human breast carcinoma MCF-7 cells were dramatically suppressed at ED₅₀ = 4.56–16.24 µg/mL → cellular apoptosis).		[64,111]
Leaves		7-O-methyl amentoflavone	Shows significant antifungal activity (especially against *Alternaria alternata* at 100 µmol/L).		[64]
Leaves, branches, bark		Sciadopitysin	Antibacterial effect; anti-Alzheimer’s activity (95% ethanolic extract: inhibits β-amyloid fibril aggregation); neuroprotective action at concentrations ranging from 0.4 to 50 μM—neuronal cell viability (SK-N-MC cells) increased at 400 μM, and cellular apoptosis was inhibited at 0.1–1 μM → this suggests potential as a novel therapeutic compound for Alzheimer’s disease; significant antifungal activity (potent inhibitory effect particularly against *Cladosporium oxysporum*, with ED₅₀ = 9 μM); as a P-glycoprotein (P-gp) and CYP3A4 inhibitor, it enhances the oral absorption of paclitaxel by limiting P-gp activity at a concentration of 50 mg/mL, while simultaneously reducing both expression and activity of CYP3A4 at 100 μg/mL.		[64,82,110,112]
Leaves, bark, branches, aril		Ginkgetin	Antibacterial and anticancer effects (inhibitory activity against HepG2 hepatocellular carcinoma cell line at 50 µmol/mL, resulting in reduced cell viability and decreased number of cancer cells); significant antifungal activity (particularly against *Alternaria alternata* at 100 µmol/L); acts as an efflux transporter inhibitor of P-glycoprotein (P-gp) and CYP3A4, enhancing the oral absorption of paclitaxel by limiting P-gp activity at a concentration of 50 mg/mL; simultaneously, it reduced both the expression and activity of CYP3A4 at a concentration of 100 µg/mL.		[64,110,113]
Leaves, branches		Amentoflavone	Antibacterial and anti-leishmanial effects (IC₅₀ = 28.5 ± 2 µmol/L, inducing mitochondrial disruption in *Leishmania amazonensis*); antiviral activity (inhibitory effect against SARS-CoV-2); significant antifungal activity. Acts as an efflux transporter inhibitor of P-glycoprotein (P-gp) and CYP3A4, enhancing the oral absorption of paclitaxel by limiting P-gp activity at a concentration of 50 mg/mL; simultaneously, it reduced both the expression and activity of CYP3A4 at a concentration of 100 µg/mL.		[64,110,114,115]
Leaves, branches		Sequoiaflavone	Antibacterial activity; functions as an efflux transporter inhibitor of P-glycoprotein (P-gp) and CYP3A4, enhancing the oral absorption of paclitaxel by limiting P-gp activity at a concentration of 50 mg/mL; simultaneously, it reduced both the expression and activity of CYP3A4 at a concentration of 100 µg/mL.		[64,110]
Leaves, branches		Isoginkgetin	Exhibits anti-inflammatory and anticancer activity (apparent inhibitory effect on A549 lung cancer cells at concentrations ranging from 2.5 to 20 µmol/L).		[64,116]
Leaves, branches		Putraflavone	Anti-inflammatory activity (inhibits reactive oxygen species production and CD69 expression).		[64,117]
Leaves		Sotetsuflavone	Anticancer activity (apparent inhibitory effect on A549 lung cancer cells at 200 mmol/L, associated with increased E-cadherin expression and decreased N-cadherin expression).		[64,118]
Leaves, branches		Kayaflavone	Antiviral activity (inhibitory effect against SARS-CoV-2).		[64,115]
Leaves, branches	Flavonols	Kaempferol	Antioxidant activity (0.5–32 µg/mL); antiviral effects (strong antiviral activity through inhibition of HIV-1 reverse transcriptase at 100 µg/mL); anti-inflammatory properties.		[64,105,119]
Leaves		Myricetin	Antiviral activity (limited effect against infectious bronchitis virus at 100 µmol/mL, with approximately 50% viral activity reduction observed at 10 µmol/mL).		[64,120]
Leaves, bark, branches		Quercetin	Antioxidant, anti-inflammatory, and antiallergic activity (intravenous administration: inhibits mast cell degranulation); hepatoprotective effect.		[64,83,105,121]
Leaves, bark		Isorhamnetin	Antiviral activity (suppressed the growth and invasion of SARS-CoV-2 in the human body).		[64,122]
Leaves		Aromadendrin	Neuroprotective effect (enhanced cell viability at 20 µmol/L and increased confluency at 2 mmol/L in SH-SY5y neuronal cells).		[64,123]
Leaves		Fisetin	Significantly reduces renal hypertrophy and albuminuria in diabetic mouse models, primarily by inhibiting the progression of glycation; shows beneficial effects in diabetes mellitus.		[64]
Leaves, branches	Flavonol glycosides	Kaempferol-3-O-rutinoside	Antioxidant effect (hydroxyl radical scavenging activity, IC₅₀ = 351.46 ± 2.30 µg/mL); potent anti-aging effect (hyaluronidase inhibition at IC₅₀ = 84.07 ± 10.46 µg/mL).		[64,124]
Leaves, branches		Kaempferol-7-O-glucoside	Antiviral activity (strong antiviral effects through inhibition of HIV-1 reverse transcriptase at 100 µg/mL).		[64,119]
Leaves, branches		Quercetin-3-O-rutinoside (rutin)	Anticancer activity (radioprotective effect on intestinal cancer by modulating ROS levels and antioxidant proteins, and inhibiting inflammasome activation at a dose of 10.25 mg/kg).		[64,125]
Leaves		Quercetin-7-O-glucoside	Antiviral activity (strong inhibitory effect against influenza virus strains at IC₅₀ = 3.1–8.19 µg/mL, associated with reduced ROS levels and suppression of virus-induced autophagy).		[64,126]
Leaves		Quercetin-3-O-α-L-arabinopyranosyl-β-D-glucopyranoside	Antidiabetic effect (enhances glucose uptake and glycogen synthesis at 20 µmol/L).		[64,127]
Leaves, branches		Quercetin-3-O-glucoside	Anticancer activity (significant antitumor effects against MCF-7 and HeLa cancer cell lines with IC₅₀ values of 36.4 µmol/L and 52.5 µmol/L, respectively).		[57,64]
Leaves, branches		Quercetin-3-rhamnoside	Antilipase activity (inhibits lipase activity at concentrations ranging from 0 to 3 × 10⁻⁵ mol/L).		[64,128]
Leaves	Flavanone glycosides	Prunin	Strong ROS-inhibitory activity; shows medicinal potential against UV-induced cutaneous tumorigenesis.		[66,129]
Leaves		Neohesperidin	Exhibits antioxidant, anti-inflammatory, and antiallergic activities, especifically effective in preventing immediate and delayed allergic diseases caused by mast cell degranulation.		[66,130]
Branches	Dihydroflavones	Pinocembrin	Antiviral activity (significant suppression of Zika virus invasion at IC₅₀ = 17.4 µmol/L, by reducing viral RNA and protein expression).		[64,131]
Branches		Eriodictyol	Antidiabetic effect (enhances cell viability and superoxide dismutase activity, while reducing ROS generation at concentrations of 5, 10, and 20 µmol/L in diabetic mice).		[64,132]
Branches		Butin	Antidiabetic effect (at 10 and 20 mg/kg: significantly reduces blood glucose levels, oxidative stress, and neuroinflammation; enhances neurobehavioral parameters and metabolic levels).		[64,132]
Branches		Naringenin	Anticancer activity (as nanoparticles: reduces proliferation and migration of A549 lung cancer cells); anti-inflammatory effect (nanoparticles: attenuates pro-inflammatory cytokines and their expression levels); antiallergic activity (intravenous administration: inhibits mast cell degranulation).		[64,121,133]
Branches		Pinostrobin	Promotes melanogenesis (inhibits tyrosinase activity with an IC₅₀ value of 700 µmol/L).		[64,134]
Leaves	Dihydroflavonols	Taxifolin	Antidiabetic activity (IC₅₀ = 0.038–0.647 mg/mL: inhibits α-amylase and α-glucosidase; regulates postprandial hyperglycemia); significant antioxidant and anti-inflammatory activity (40 mg/kg); chemopreventive, hepatoprotective, and cardioprotective properties (100 µg/kg/day: inhibits angiotensin II-converting enzyme and suppresses ROS formation; 3.3 mg/kg: lowers elevated blood pressure). Anti-Alzheimer’s potential (prevents and/or treats cognitive dysfunction related to β-amyloid fibril aggregation); antiviral activity (against hepatitis A virus at 59 µg/mL); antibacterial action (*Propionibacterium acnes*; *Toxoplasma gondii* in combination with pyrimethamine, IC₅₀ = 1.39 µg/mL); antilipase and antityrosinase effects; inhibits ROS production. Enhances the efficacy of ceftazidime and levofloxacin in treating methicillin-resistant *Staphylococcus aureus* (MRSA) infections; suppresses osteoclast activity and mitigates ovariectomy-induced bone loss (potential alternative to estrogen therapy). Demonstrates therapeutic potential in treating bacterial infections (including acne and toxoplasmosis), liver disorders (including autoimmune hepatitis), cardiovascular diseases, inflammation, cancer, viral infections, Alzheimer’s disease, diabetes, allergic reactions, and immune deficiencies.		[64,83,135]
Leaves, bark, branches, roots	Flavanols	(+)-Catechin	Antioxidant activity (inhibits autooxidation at 0.01 mol/L; IC₅₀ = 16.88 µg/mL compared to ascorbic acid at 14.48 µg/mL); antidiabetic effect (significantly inhibits α-amylase at IC₅₀ = 0.752 mg/mL and α-glucosidase); anticancer activity (significant antitumor effects against MCF-7, HeLa, and HepG2 cancer cell lines); hepatoprotective properties.		[57,64,83,136,137,138,139]
Leaves, bark, branches, roots		(−)-Epicatechin	Antioxidant activity (inhibits autooxidation at 0.01 mol/L; IC₅₀ = 20.20 µg/mL compared to ascorbic acid at 14.48 µg/mL); antidiabetic effect (significantly inhibits α-amylase at IC₅₀ = 0.655 mg/mL and α-glucosidase). Anticancer activity (significant antitumor effects against MCF-7, HeLa, and HepG2 cancer cell lines; reduces radiation resistance and enhances therapeutic effects by activating cyclooxygenase in pancreatic cancer cells at 200 µmol/L); also demonstrates radioprotective effects in human fibroblasts at 20 µmol/L.		[57,64,136,138,139,140,141]
Leaves		Gallocatechin	Antidiabetic and antiviral activity (suppresses SARS-CoV-2 replication with an IC₅₀ = 13.14 ± 2.081 µmol/L).		[64,139,142]
Leaves		Epigallocatechin	Antidiabetic activity.		[64,139]
Leaves		Procyanidin B2	Antidiabetic activity (protected endothelial progenitor cell function, reduced oxidative damage, and promoted wound healing and angiogenesis in diabetic mice).		[64,136,143]
Branches	Chalcones	Pinocembrin chalcone	Antibacterial activity (moderate inhibitory effect against *Neisseria gonorrhoeae* at 128 µg/mL).		[64,144]
Branches		Isoliquiritigenin	Significant hepatoprotective activity (at 10 µmol/L, reduced transaminase and inflammatory cytokine levels while enhancing catalase activity).		[64,145]
Branches		Butein	Significant anti-inflammatory and antinociceptive effects, demonstrated by reduced nociception in thermal and paw edema tests in mice at 10–20 mg/kg, accompanied by decreased levels of inflammatory cytokines.		[64,146]
Branches		Naringenin chalcone	Anti-inflammatory and antiallergic activity (intravenous administration: inhibits mast cell degranulation).		[64,121]
Leaves	Isoflavones	Glycitin (glycitein-7-O-glucoside)	Protective effect against UV-induced skin photoaging in primary human dermal fibroblasts and against lipopolysaccharide-induced acute lung injury.	Exhibits beneficial effects in the fight against cancer, in the treatment of cardiovascular diseases, as well as in the prevention and management of Alzheimer’s disease.	[66,147]
Leaves		Genistin (genistein-7-O-glucoside)	Protective effect of genistin on wild-type p53 cells against taxol-induced cytotoxicity in the combined treatment of lung cancer.		[66,148]
**LIGNANS**					
Bark, leaves	Taxiresinol		Anticancer activity (methanolic extracts).	Anticancer, antiulcer activities.	[25,26,65,80]
Roots	(−)-7-O-methyltanegool		α-glucosidase inhibitory effects.		
Roots	13α-Conidendrin				
Roots	4-Formosanol				
Roots	14(+)-Tsugacetal				
Roots	14α-Intermedianol				
Roots	14-Oxabicyclooctalignan				
Roots	Lanceolatanin C				
Roots	Lanceolatanin D				
Roots	Matairesinol				
Roots	147-Methoxymatairesinol				
Roots	17-Oxomatairesinol				
Bark	Taxiresinol I		Ethanolic extract exhibits anticancer activity (in vitro) against ovarian, colon, breast, and liver cancer cells; also shows anti-inflammatory and antinociceptive properties; antiulcer effects; and antifungal activity against *Nigrospora oryzae*, *Epidermophyton floccosum*, *Curvularia lunata*, and *Pleurotus ostreatus*.		
Bark	Isotaxiresinol		Exhibits therapeutic potential in postmenopausal osteoporosis by promoting bone formation and inhibiting bone resorption.		
Bark	Lariciresinol		Exhibits anti-inflammatory and antinociceptive properties, as well as antifungal activity against *Nigrospora oryzae*, *Epidermophyton floccosum*, *Curvularia lunata*, and *Pleurotus ostreatus*.		[38,68,80]
Bark	Isolariciresinol				
Bark	30-demethyl-isolariciresinol-9′-hydroxyisopropyl ether				
Bark	3-demethyl-isolariciresinol				
**PHYTOSTEROLS**					
Bark	Daucosterol		Exhibits cytotoxic activity against various cancer cell lines, including HepG2 (liver cancer), MCF-7 (breast cancer), HeLa (cervical cancer), and A549 (lung cancer).		[26,37,65]
Bark, seeds	β-Sitosterol		Exhibits antiviral, anti-inflammatory, antipyretic, and uterotrophic effects.		

**Table 5:** Overview of Patent-Derived Dermato-cosmetic Formulations Incorporating *Taxus* Cambial and Procambial Cell Extracts [161].

**Body Lotion Based on *Taxus* Cambium and Procambium**	**Cream Based on *Taxus* Cambium and Procambium**
◆ 6.2 mg cell extract from the invention line ◆ 6.5 mg 1,3-butylene glycol ◆ 1.2 mg glycerol ◆ 0.2 mg D-panthenol ◆ 0.3 mg ethyl alcohol ◆ 0.1 mg carbomer ◆ 1.5 mg stearic acid ◆ 0.7 mg polysorbate 60 ◆ 0.6 mg lipophilic glyceryl stearate ◆ 0.3 mg sorbitan sesquioleate ◆ 0.6 mg cetearyl alcohol ◆ 3.5 mg squalene ◆ 3 mg caprylic/capric acid triglycerides ◆ 0.4 mg dimethicone ◆ a small amount of preservative ◆ the desired amount of perfume and purified water in an amount such that the total weight of the preparation is 100 mg.	◆ 5.0 mg cell extract from the invention line ◆ 7.0 mg 1,3-butylene glycol ◆ 1.0 mg glycerol ◆ 0.1 mg D-panthenol ◆ 0.4 mg magnesium aluminosilicate ◆ 2.0 mg stearic acid ◆ 1.5 mg polysorbate 60 ◆ 2.0 mg lipophilic glyceryl stearate ◆ 1.5 mg sorbitan sesquioleate ◆ 4.0 mg mineral oil ◆ 3.0 mg cetearyl alcohol ◆ 3.8 mg squalene ◆ 2.8 mg caprylic/capric acid triglycerides ◆ 0.4 mg dimethicone ◆ the required amount of xanthan gum ◆ the required amount of triethanolamine ◆ the required amount of tocopherol acetate ◆ a small amount of preservative ◆ the desired amount of perfume and purified water in such a quantity that the total weight of the preparation is 100 mg.
The formulations described in the present invention demonstrate efficacy in the prevention and delay of skin aging. Furthermore, these formulations exhibit notable depigmenting and anti-inflammatory properties.	

**Table 4:** Therapeutic Effects of the Aril of *Taxus* Species.

Type of Extract/Chemical Compound	Therapeutic Action	References
Hydro-methanolic extract	Exhibits antibacterial activity against *Pectobacterium* sp. and *Dickeya chrysanthemi*, with a minimum inhibitory concentration (MIC) ranging from 1000 to 1500 µg/mL.	[33]
Ethanolic Extract (Rhodoxanthin)	Demonstrated significant antiproliferative and pro-apoptotic effects on murine melanoma B16F10 cells in vitro. Oral administration of 7 mg rodoxanthin/kg body weight for 21 days significantly reduced tumor growth (by 42.18%) and tumor weight (by 15.74%) in CD57BL/6J mice bearing B16F10 melanoma; also significantly increased erythrocyte count, hemoglobin, and hematocrit levels in treated tumor-bearing mice. Showed dose-dependent in vitro cytotoxic activity on metastatic murine melanoma B16F10 cell line—the highest tested concentration (C_1_ = 0.18 µmol/mL) reduced cell viability to 32.29%, while the lowest concentration (C_5_ = 0.025 µmol/mL) reduced viability to 48.58%, indicating an inverse correlation between rodoxanthin concentration and melanoma cell viability. Exhibited cytoprotective effects against hydrogen peroxide-induced oxidative stress in human HaCaT epidermal keratinocytes and human retinal epithelial cells, improving cell viability by 12.55%, 13%, and 9.66%, respectively.	[34,80]
Ethanolic Extract (Phenolic Compounds)	Exhibits moderate antioxidant activity (IC₅₀ = 68.46 mg/mL), with an antioxidant capacity (10.7 µmol/100 mL) comparable to that of blackcurrant fruits (*Ribes nigrum*) (10 µmol/100 mL).	[77]
Methanolic Extract	Exhibits antifungal activity against *Candida albicans* and *Aspergillus brasiliensis*.	[80]
Methanolic, Acetone, and Distilled Water Extracts	Antioxidant activity (the methanolic extract showed the highest potency, followed by the distilled water extract; the acetone extract was the least effective). Hypoglycemic activity (inhibition of α-amylase and α-glucosidase)—the methanolic extract was the most potent, followed by the distilled water extract, with the acetone extract showing the weakest effect.	[30]
Carotenoids	Reduce the harmful effects of reactive oxygen species (ROS), contributing to the prevention and mitigation of degenerative diseases, macular degeneration, cardiovascular conditions, and several types of cancer, including lung, gastric, pancreatic, breast, and prostate cancers.	[77]
Terpenoids	Exhibit antimicrobial activity (limonene, p-cymene, α/β-pinene), anti-inflammatory and chemopreventive effects (limonene), antioxidant and hepatoprotective properties, as well as antiulcer activity (α-pinene, limonene) and antispasmodic action (α-pinene).	[31,84]
Ferulic acid, p-Coumaric acid, Caffeic acid	Exhibit immunomodulatory, cytoprotective, and antioxidant effects.	[31,32]
Ascorbic Acid	The aril of *Taxus* species provides the full recommended dietary allowance (RDA) of vitamin C for healthy adults.	[31,32]
Carbohydrates	A total of 100 g of aril yields approximately 106 kilocalories, making it suitable as a low-calorie snack option.	[31,32]
Amino Acids	The aril can serve as a high-quality protein source in human nutrition, containing 43% essential amino acids (according to WHO, foods with over 40% essential amino acids are considered ideal protein sources). The content of branched-chain amino acids (leucine, isoleucine, valine) in the aril (18.4%) is comparable to that found in animal-derived proteins (20%).	[31,32]
Macroelements and Microelements	The potassium content of the aril is comparable to that of bananas, which are widely considered a typical dietary source of potassium. A 100 g serving of aril provides 11–15% of the recommended daily intake of zinc, 9–15% of iron, and 30–50% of chromium.	[31,32]
Aril Juice	It alleviates Alzheimer’s disease by modulating several biological processes, including oxidative stress, inflammatory responses, neuronal apoptosis, insulin secretion, amyloid fibril formation, and T-cell co-stimulation.	[88,149]

## Data Availability

There are no additional data to be published.

## Supplementary Materials

The following supporting information can be downloaded at: https://www.mdpi.com/article/10.3390/plants14101439/s1, Table S1: Traditional Uses of *Taxus* Species Around the World.

## Abbreviations

The following abbreviations are used in this manuscript:

ADA	Adenosine Deaminase
Al	Aluminum
Cd	Cadmium
DNA	Deoxyribonucleic Acid
ED₅₀	Median Effective Dose
FDA	Food and Drug Administration
GC-MS	Gas Chromatography–Mass Spectrometry
GLUT4	Glucose Transporter Type 4
GPX4	Glutathione Peroxidase 4
HDL	High-Density Lipoprotein
HIV	Human Immunodeficiency Virus
HPLC-MS/MS	High-Performance Liquid Chromatography–Tandem Mass Spectrometry
IC₅₀	Half-Maximal Inhibitory Concentration
IL-1β	Interleukin 1-Beta
IUCN	International Union for Conservation of Nature
LDL	Low-Density Lipoprotein
MMP-1	Matrix Metalloproteinase 1
MRSA	Methicillin-Resistant *Staphylococcus aureus*
NCI	National Cancer Institute
Ni	Nickel
NSCLC	Non-Small Cell Lung Cancer
P-gp	P-Glycoprotein
PMI-FAs	Polymethylated Fatty Acids
RDA	Recommended Dietary Allowance
RNA	Ribonucleic Acid
ROS	Reactive Oxygen Species
SARS-CoV-2	Severe Acute Respiratory Syndrome Coronavirus 2
SOD	Superoxide Dismutase
TNF-α	Tumor Necrosis Factor-Alpha
UPLC-ESI-MS/MS	Ultra-Performance Liquid Chromatography Coupled with Electrospray Ionization Tandem Mass Spectrometry
UV	Ultraviolet
WHO	World Health Organization
