# Supplementary material for: A Deep Dive into the Botanical and Medicinal Heritage of Taxus
Source: Plants (Basel). 2025 May 11;14(10):1439. doi: 10.3390/plants14101439 (PMC12115136; doi:10.3390/plants14101439)
Supplement: Supplementary file 1 [file plants-14-01439-s001.zip › plants-3592977-supplementary.pdf]

plants

MDPI

Supplementary Material

Table S1. Traditional Uses of *Taxus* Species Around the World.

Species and Region:	Plant Part Used:	Preparation / Administration:	Traditional Uses:	References:
Taxus wallichiana (India – Bhotiya tribe)	Bark	Tea: the bark is powdered, boiled in water, and mixed with milk, ghee, and salt to make tea	✓ to keep the body warm;<br>✓ treatment of hemorrhoids.	[26]
		Decoction: a concentrated liquid prepared by boiling the bark powder in water	✓ treatment of muscle and joint pain;<br>✓ treatment of rheumatism.	
		Decoction with jaggery: a decoction is prepared from the bark, filtered, mixed with jaggery, and administered for 14 days	✓ treatment of hysteria.	
		Paste made from the powdered bark	✓ for the healing of fractured bones;<br>✓ treatment of headaches by applying it to the forehead.	
	Young branches	Tincture: an alcoholic extract	✓ prevention of headaches, dizziness, diarrhea, and weak pulse.	
		Decoction: a concentrated liquid prepared by boiling the powder of young buds in water	✓ treatment of tuberculosis.	
	Leaves	Decoction/juice: a concentrated liquid prepared by boiling the leaves in water	✓ for liver disorders;<br>✓ treatment of asthma, cancer, and bronchitis.	
		Powder from dried leaves	✓ treatment of asthma, bronchitis, hiccups, epilepsy, diarrhea, and headaches.	

		Extract/juice	✓ for the treatment of cuts, wounds, and boils (external use);<br>✓ sedative;<br>✓ as an antidote against snake bites and scorpion stings.	
Taxus wallichiana (India)	Bark, seeds	Extract from bark and seeds mixed with warm water (oral)	✓ treatment of intestinal parasites.	[37]
	Bark	Bark extract prepared as a tea	✓ treatment of high blood pressure, asthma, headaches, dizziness, and tumors.	
		Tea: a preparation of powder with boiling water	✓ treatment of colds, asthma, arthritis, dizziness, and tumors.	
		Paste made from bark powder	✓ for healing fractured bones, treating headaches, and hemorrhoids.	
		Dried bark powder, mixed with salt and ghee, is blended with water to make tea	✓ treatment of high blood pressure and cancer.	
		Bark paste mixed with egg yolk	✓ used as a plaster for healing fractured bones.	
		Decoction	✓ treatment of cancer.	
	Leaves	Juice from the leaves	✓ treatment of wounds, cuts, and boils.	
		Decoction	✓ treatment of asthma, bronchitis, and colds.	
		Decoction of leaves with honey	✓ treatment of fever, flatulence, epilepsy, and asthma.	
		Tea	✓ treatment of asthma and fever.	
	Bark, leaves	Tea	✓ treatment of cancer and tumors;<br>✓ treatment of swellings;<br>✓ used as a contraceptive measure;<br>✓ treatment of congestion, cough, and asthma.	
	Young branches	Tincture	✓ treatment of headaches, dizziness, weak pulse, diarrhea, and severe biliary disorders.	

	Stem	Decoction	✓ treatment of tuberculosis.	
	The entire plant	—	✓ treatment of cancer, jaundice, heart disorders, headaches, renal and digestive disorders;<br>✓ exhibits antispasmodic, laxative, and antirheumatic effects.	
Taxus baccata (India – Pauri district, Uttarakhand)	Bark, leaves	—	✓ treatment of headaches, bone fractures, cancer, asthma, bronchitis, epilepsy, arthritis, and snake bites.	[56]
Taxus contorta, Taxus mairei, Taxus wallichiana (Hindu Kush-Himalayan region)	Arils	Consumed as is	✓ used as a snack.	[35]
Taxus contorta, Taxus mairei, Taxus wallichiana (Hindu Kush-Himalayan region)	Leaves	Juice from leaves with honey	✓ cleanses the respiratory tract and helps treat cough and colds.	
Taxus contorta, Taxus mairei, Taxus wallichiana (Hindu Kush-Himalayan region)	Leaves	¼ cup of leaf juice or decoction (oral)	✓ treatment of diarrhea, indigestion, stomach pain, and liver disorders.	
	Aril	Consumed as is	✓ has carminative, expectorant, and stomachic properties.	
	Leaves, bark	Juice from leaves and bark (2 teaspoons), consumed daily	✓ treatment of fever, low pulse, and cancer.	
	Bark	Tea	✓ used to enhance virility.	
Taxus wallichiana (Hindu Kush-Himalayan region)	Leaves, bark	A paste is made from the leaves and bark and administered with honey	✓ treatment of bronchitis, asthma, and other respiratory problems.	
Taxus contorta, Taxus wallichiana (Hindu	Leaves	Leaf juice (oral)	✓ treatment of headaches.	
	Bark	Paste made from bark powder (external use)	✓ applied on the forehead for headache relief.	

Kush-Himalayan region)		Decoction	✓ treatment of muscle and joint pain, and rheumatism.	
Taxus wallichiana	—	Decoction, tea, juice	✓ treatment of colds, cough, respiratory infections, indigestion, and epilepsy.	[49,57]
	—	Poultice	✓ treatment of burns and infected wounds.	
	Bark	Paste made from bark powder	✓ treatment of bone fractures and headaches.	
	Bark, leaves	Steam baths	✓ treatment of rheumatism.	
	Stem	Decoction – in Pakistan	✓ treatment of tuberculosis.	
	Bark, leaves	Unani Medicine	✓ sedative, aphrodisiac;<br>✓ treatment for bronchitis, asthma, epilepsy, snake bites, and scorpion stings.	
	Young branches	Ayurvedic tincture	✓ used to treat severe biliary disorders, dizziness, weak pulse, cold extremities, headaches, and diarrhea.	
Taxus baccata	—	Asturias, León	✓ treatment of rheumatism, arthritis, liver disorders, and urinary tract disorders.	[5]
	Aril	Aril pulp – syrup (Northern Spain)	✓ treatment of pulmonary disorders.	
Taxus wallichiana (Indian Ayurvedic Pharmacopoeia)	Leaves	Powder obtained from dried leaves	✓ with antirheumatic, anticatarrhal, and insecticidal action;<br>✓ wound healing;<br>✓ treatment of tumors, dermatoses, and helminthiasis.	[26]
Taxus baccata (Ukraine, Bukovina)	Bark	Bark decoction	✓ treatment against rabies.	[4]
Taxus baccata (Narew River, Northeast Poland)	Bark	Bark powder, transformed into a dough with rye flour	✓ treatment against rabies.	
Taxus baccata (Central Balkan Peninsula)	Bark	Crushed bark	✓ treatment of rabies, epilepsy, and tuberculosis.	

Taxus baccata (Poland – Wólka Jagielczynska village, Częstochowa)	Bark	Infusion with milk or fumigation of the wood	✓ treatment of rabies.
